# The HXD95: a modified Bassett-type hydrothermal diamond-anvil cell for *in situ* XRD experiments up to 5 GPa and 1300 K

**DOI:** 10.1107/S1600577519016801

**Published:** 2020-01-29

**Authors:** Marion Louvel, James W. E. Drewitt, Allan Ross, Richard Thwaites, Benedict J. Heinen, Dean S. Keeble, Christine M. Beavers, Michael J. Walter, Simone Anzellini

**Affiliations:** a School of Earth Science, University of Bristol, Wills Memorial Building, Queens Road, Bristol BS8 1RJ, UK; b Institute for Mineralogy, WWU, D-48149 Munster, Germany; c Diamond Light Source Ltd, Harwell Science and Innovation Campus, Diamond House, Didcot OX11 0DE, UK; d Geophysical Laboratory, Carnegie Institution for Science, 5251 Broad Branch Road NW, Washington, DC 20015, USA

**Keywords:** diamond-anvil cells, resistive heating, synchrotron characterization, metallic liquids, glasses and melts, extreme conditions

## Abstract

A new diamond-anvil cell apparatus for *in situ* synchrotron X-ray diffraction measurements of liquids and glasses, at pressures from ambient to 5 GPa and temperatures from ambient to 1300 K, is reported. This portable setup enables *in situ* monitoring of the melting of complex compounds and the determination of the structure and properties of melts under moderately high pressure and high temperature conditions relevant to industrial processes and magmatic processes in the Earth’s crust and shallow mantle. The device was constructed according to a modified Bassett-type hydro­thermal diamond-anvil cell design with a large angular opening (θ = 95°). This paper reports the successful application of this device to record *in situ* synchrotron X-ray diffraction of liquid Ga and synthetic PbSiO_3_ glass to 1100 K and 3 GPa.

## Abstract 2

A Bassett-type hydro­thermal diamond-anvil cell has been modified to enable *in situ* X-ray diffraction measurements on liquid samples up to 5 GPa and 1300 K at the Diamond Light Source. The new experimental setup provides a maximum accessible scattering vector *Q*_max_ of 18 Å^−1^ (at 56 keV) for improved resolution in *G*(*r*).

## 1. Introduction   

Understanding the structural response in liquids and glasses at elevated pressure (*P*) and temperature (*T*) is important from technological, geological and fundamental scientific perspectives. Many engineering applications, from civil aviation to power generation facilities, require materials to operate at increasingly extreme conditions of *P* and *T*. Understanding the response of alloys and glasses at moderately high *P* and/or *T* is important for monitoring material damage under high mechanical loads during industrial production processes, or under the extreme conditions experienced in nuclear power generation facilities (Ding *et al.*, 2018 ▸; Fuhrmann *et al.*, 2014 ▸; Nie & Chen, 2013 ▸; Steinbrück & Böttcher, 2011 ▸). In the Earth’s crust, the compositional and structural changes that magmas experience upon cooling and decompression affect their viscosity and may ultimately control their eruptive behaviour, even on short timescales (*e.g.* the transition from ‘quiet’ effusive versus ‘cataclysmic’ explosive eruptions; Di Genova *et al.*, 2017 ▸). Structural changes associated with fractional crystallization of magmas further affect trace-element partitioning between melts and crystals and a better understanding of the local environment in silicate melts is necessary to build accurate geochemical models of magmatic processes throughout geological times (Corgne *et al.*, 2012 ▸; Gaetani, 2004 ▸; Prowatke & Klemme, 2005 ▸).

The structure of liquids and glasses cannot be described in terms of a periodically repeating unit cell as for crystalline solids. As such, it is inherently difficult to characterize the atomic scale structure of liquids, although chemical bonding constraints can lead to a high degree of ordering on short- and intermediate-length scales, which can be readily revealed by nuclear magnetic resonance (NMR), Raman spectroscopy or neutron and synchrotron X-ray diffraction (XRD) methods. Diffraction provides a direct measure of the atomic scale structure of liquids and glasses in the form of the total pair distribution function (PDF), denoted *G*(*r*), describing the probability of finding two atoms separated by distance *r*. In a synchrotron XRD experiment, far from an absorption edge on a polyatomic liquid or glass containing *n* chemical species, the coherent scattering is represented by the total structure factor *S*(*Q*) as comprised from the weighted sum of *n*(*n* + 1) Faber–Ziman partial structure factors *S*_αβ_(*Q*):
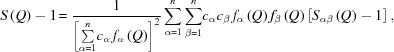
where *c* and *f* denote the atomic fractions and X-ray form-factors of chemical species α or β, respectively, and *Q* is the scattering vector. The corresponding total PDF *G*(*r*) comprises a weighted sum of *n*(*n* + 1)/2 overlapping partial pair-distribution functions *g_αβ_*(*r*) representing the atom–atom correlations and is obtained by the Fourier transform relation:
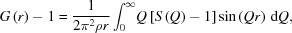
where ρ is the atomic number density (Kohara & Salmon, 2016 ▸; Benmore, 2012 ▸).

The structural response of liquids and glasses to high *P* may involve changes in bond lengths and angles, changes in the nearest-neighbour coordination environments, and a reorganization of the connectivity between these local coordination polyhedra. The technical demands inherent in measuring quality diffraction data suitable for PDF analysis have been discussed in depth previously (Chupas *et al.*, 2003 ▸; Fischer *et al.*, 2006 ▸); however, measuring changes in the disordered structure of liquids and glasses at high *T* and/or high *P* is doubly challenging, requiring specialized instrumentation to generate these extreme conditions while providing good accessibility to the sample and minimizing unwanted contributions from the sample environment to reliably extract the diffuse liquid sample signal. As a result of these experimental challenges, ambient-*T* glass analogues have long been used to approximate liquid structure at high *P*–*T* (Henderson, 2005 ▸). However, recent advances in both high-*T* and high-*P* experimental and analytical techniques now allow for the structure and properties of liquids to be measured *in situ* under extreme conditions (Kono *et al.*, 2014 ▸; Morard *et al.*, 2014 ▸). Such measurements provide a rigorous check on the efficacy of first principles or classical molecular dynamics simulations which, in turn, can provide a detailed description of the three-dimensional atomistic structure of molten alloys and silicate melts to high *P* (Bajgain *et al.*, 2015 ▸; Drewitt *et al.*, 2015 ▸; Le Losq *et al.*, 2017*a* ▸; Moussallam *et al.*, 2016 ▸).

The ambient-*P* structure and dynamics of high-*T* liquid oxides and metals can be reliably determined to >3000 K by combining container-less techniques (*e.g.* aerodynamic or electrostatic levitation with laser heating) with NMR, neutron diffraction or high-energy XRD (Hennet *et al.*, 2011 ▸; Benmore & Weber, 2017 ▸). The advantages of container-less processing include the prevention of potential chemical reactions between the sample and its container that can occur in conventional high-*T* furnaces and very low contributions to the measured signal from the sample environment. Recent studies include the structure of levitated liquid Al–Cu and Al–Ni alloys (Brillo *et al.*, 2006 ▸), *in situ* high-*T* ^27^Al NMR of levitated SrO–Al_2_O_3_–SiO_2_ liquids (Florian *et al.*, 2018 ▸), iron coordination environments in FeO–SiO_2_ liquids (Drewitt *et al.*, 2013 ▸; Alderman *et al.* 2017 ▸), and *in situ* neutron diffraction with isotope substitution (NDIS) to reveal detailed structural information in levitated Ni_36_Zr_64_ (Voigtmann *et al.* 2008 ▸), Ni–Si (Gruner *et al.* 2009 ▸), CaO–Al_2_O_3_ (Drewitt *et al.*, 2012 ▸; Drewitt *et al.*, 2017 ▸) and CaSiO_3_ (Skinner *et al.*, 2012 ▸) liquids on a partial PDF level.

Several different approaches are available to investigate the structure and properties of liquids at simultaneous high *P* and high *T*. Large-volume presses, including the Paris–Edinburgh (P–E) cell, equipped with resistive heaters, installed at energy-dispersive synchrotron beamlines provide a firmly established method for making XRD measurements of silicate melts at pressures up to 10 GPa (Funamori, 2004 ▸; Yamada *et al.*, 2011 ▸; Sakamaki *et al.*, 2012 ▸; Sanloup *et al.*, 2013*a* ▸; Wang *et al.*, 2014 ▸; Kono *et al.*, 2014 ▸). Nevertheless, these experiments remain challenging, particularly for measurements involving low-viscosity samples such as hydrous melts, which can readily escape the P–E assembly (Malfait *et al.*, 2014 ▸; Ritter *et al.*, 2017 ▸). In a P–E cell, temperature is typically estimated from heater-power calibration curves and is not usually measured *in situ*, giving rise to large uncertainties in temperature on the order of 13% (Crichton & Mezouar, 2005 ▸). An alternative approach involves using a laser-heated diamond-anvil cell (LH-DAC) and angular-dispersive (AD) synchrotron XRD. AD-XRD enables faster data acquisition times (from a few seconds to several minutes) compared with the several hours for energy-dispersive XRD. The LH–DAC method enables *P* up to the Mbar range with *T* between 1600 K and 5000 K, where *T* is measured *in situ* by spectral radiometry (Walter & Koga, 2004 ▸). The LH–DAC has been widely used to investigate solid–solid phase relations, high-*P* melting curves, and the structure of pure-metal and geological melts *in situ* up to 5000 K and 50–200 GPa (Watanuki *et al.*, 2001 ▸; Shen *et al.*, 2004 ▸; Anzellini *et al.*, 2013 ▸, 2018 ▸ *b*, 2019 ▸ *a*; Sanloup *et al.*, 2013*b* ▸; Lord *et al.*, 2014 ▸; Morard *et al.*, 2014 ▸; Drewitt *et al.*, 2015 ▸, 2019 ▸; McGuire *et al.*, 2017 ▸). Simultaneous high-*P*–*T* conditions in the DAC can also be achieved using resistive heating (RH) (Kantor *et al.*, 2012 ▸; de Grouchy *et al.* 2017 ▸). Although the maximum *T* achievable in an RH–DAC is typically lower than in the LH–DAC, the method has the significant advantage of providing homogeneous heating over the whole sample. This homogeneity also allows for larger sample volumes and beam sizes to be used, which is key for increasing signal-to-noise when utilizing high-energy X-rays.

Regardless of the approach (P–E or LH/RH–DAC), measuring disordered liquid or glass structures at high-*P in situ* by XRD is challenging due to the requirement to accurately characterize the *P*-dependent scattering from the high-*P* cell assembly for reliable extraction of the diffuse sample signal. To overcome this problem, spatial collimation may be employed to reduce the measured scattering contribution from the pressure vessel. With energy-dispersive XRD, the scattering is measured at a fixed angle. Certain background features, such as crystalline Bragg diffraction peaks, can be eliminated by collecting data at several 2θ angles and combining the different energy-dispersive data sets by normalization of the intensity to the X-ray source spectrum. For AD–XRD measurements, multichannel collimators may be employed (Yaoita *et al.*, 1997 ▸; Mezouar *et al.*, 2002 ▸). Alternatively, modifications can be made to the design of the pressure vessel to reduce the fraction of non-sample components within the primary X-ray path. For example, most of the Compton scattering from the diamond anvils can be eliminated by using perforated diamonds (Soignard *et al.*, 2010 ▸; Chapman *et al.*, 2010 ▸). Consideration should also be made to maximize the accessible scattering vector *Q* range in order to provide good resolution in *G*(*r*), determined from the Fourier transform of the measured structure factor *S*(*Q*). At high *P*, *Q*_max_ is often limited by the opening angle of the cell and the photon energy used in the diffraction experiment; high-energy synchrotron X-rays are essential to achieving a satisfactory *G*(*r*) from a high-*P* diffraction experiment.

The resistive elements in an RH–DAC device can be placed either externally to heat the entire DAC or internally within the DAC surrounding the diamond anvils. Externally heated designs are limited to *T* < 900 K, *i.e.* below the melting conditions of most glasses and magmas (Stinton *et al.*, 2014 ▸; Anzellini *et al.*, 2018 ▸ *a*). Although internally heated designs are operational in the *T* range 300–1300 K (Fei & Mao, 1994 ▸; Pasternak *et al.*, 2008 ▸; Du *et al.*, 2013 ▸), and some designs suitable for liquid and glass RH–DAC XRD experiments already exist (Kantor *et al.*, 2012 ▸; de Grouchy *et al.* 2017 ▸), a device specifically optimized for routine liquid diffraction in the moderately high-*P* and high-*T* conditions relevant to industrial, environmental and crustal magmatic processes is acutely needed. The operational *P*–*T* range of the hydro­thermal diamond-anvil cell (HDAC) developed by Bassett *et al.* (1993 ▸, 2000 ▸) at the end of last century can reliably achieve these conditions and has been successfully used in combination with Raman spectroscopy and synchrotron radiation techniques (X-ray fluorescence, absorption, small-angle X-ray scattering) to study high-*P*–*T* fluids and melts of geological interest up to 1300 K and 5 GPa (Bassett *et al.*, 2000 ▸; Bureau *et al.*, 2007 ▸; Facq *et al.*, 2016 ▸; Le Losq *et al.*, 2017*b* ▸; Louvel *et al.*, 2013 ▸; Mayanovic *et al.*, 2007 ▸; Wilke *et al.*, 2006 ▸). However, the HDAC is unsuitable for synchrotron XRD due to its low angular access to the sample (Bassett *et al.*, 1993 ▸).

In this paper, we describe the construction of the HXD95 device, a new RH–DAC system based on a modified Bassett design but optimized with large opening Boehler–Almax seats for *in situ* synchrotron XRD measurements of liquids and glasses at variable-*P* conditions from ambient to 5 GPa and *T* from ambient to 1300 K. We provide a technical description of the new cell and the synchrotron XRD setup, and report two example measurements made in the device of the structure of liquid Ga and high-*P* melting of synthetic PbSiO_3_ glass.

## 2. Experimental setup   

### 2.1. Technical description of the modified Bassett HDAC   

The HXD95 RH–DAC is based on a modified Bassett-type design (HDAC-III; Bassett, 2003 ▸), optimized for *in situ* synchrotron XRD measurements of liquids and glasses within the specific moderately high *P*–*T* domain relevant to industrial and crustal magmatic processes. The Bassett-type resistive heater design, in which heat from the resistive elements is transmitted to the sample through both diamond anvils, is suited for routine operational usage up to 1100 K, with higher-*T* heating reported up to 1300 K (Audétat & Keppler, 2005 ▸; Le Losq *et al.*, 2017*a* ▸; Louvel *et al.*, 2013 ▸; Mayanovic *et al.*, 2007 ▸). Advantages of the Basset-type design include: (i) a simple ‘in-house’ mounting procedure, where all parts and connections, including the heating elements, can be prepared by the user from affordable components; (ii) easy diamond alignment facilitated by direct access to both diamond seats when the cell is closed; and (iii) the ability to perform high-*T* operations directly in air by flushing an inert Ar–H_2_ gas (98%-2%) inside the cell to prevent oxidation of heaters, WC seats and diamond anvils. While vacuum chambers are frequently used to prevent oxidation of RH–DACs (Stinton *et al.*, 2014 ▸), these configurations introduce several disadvantages such as (i) increasing the background elastic and inelastic scattering and fluorescence contributions making it more difficult to extract a clean low-noise signal from the pressurized sample, (ii) limiting the smallest accessible sample-to-detector distance and exit-aperture opening, thereby reducing the maximum accessible scattering vector *Q*_max_ and hence reducing the resolution in *G*(*r*), and (iii) increasing the sample changing time. With the HDAC-III, high-*T* is achieved by passing a current through a 250 µm-thick Mo wire coil wound around the WC seats which support the diamond anvils. Pressure is transmitted to the sample through a set of three driver screws equipped with pairs of Belleville washers, allowing *P* up to 3–5 GPa, depending on the diamond-anvil culet size. Further information on the original cell design and later modifications for different spectroscopic techniques and collection angles (*i.e.* 90° from the incoming beam) can be found in Bassett *et al.* (1993 ▸, 2000 ▸), Bassett (2003 ▸) or Li *et al.* (2016 ▸).

**Figure 1:**
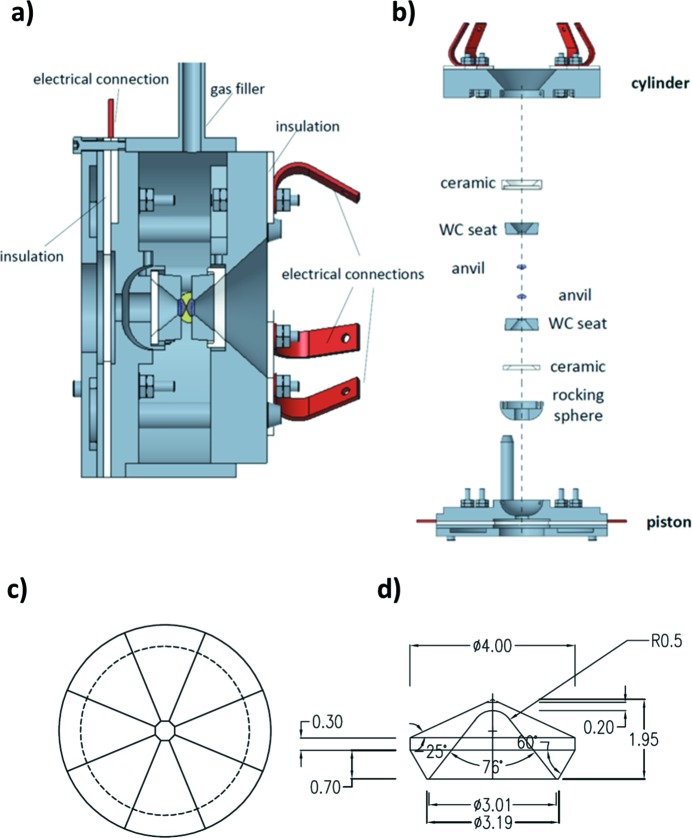
CAD-drawing of the new HXD95 cell: (*a*) lateral section and (*b*) exploded figure. The heaters (not drawn) are constructed from 250 µm-diameter Mo wire wound around the WC seats. (*c*) Top and (*d*) side view of the CAD-drawing of the specially perforated Almax–Boehler diamond anvil used during the experiments on the downstream side of the cell. The units in (*d*) are degrees and millimetres.

The original HDAC-III design employs WC anvil seats with 60° opening and standard cut diamonds (Bassett *et al.*, 1993 ▸) that do not enable a sufficiently large *Q*_max_ for good resolution in *G*(*r*). To enable a larger collection angle and improved *Q*_max_, the new HXD95 RH–DAC has been equipped with Almax–Boehler seats and diamond anvils (Boehler, 2006 ▸). The cell has been modified in order to accommodate the larger Boehler seats, and to maximize the downstream opening angle for large-angle diffraction measurements. As for the original model, the heaters are constructed with Mo wire wound around the WC seats, with the wires glued in place by embedding them in fast cure electrically insulating alumina cement (Cotronics Resbond 940 HT Powder). The heaters/seats are then glued onto specially designed zirconia–alumina ceramics, manufactured by Precision Ceramics to reduce thermal dispersion and electrically insulate the heaters from the cell body (see Figs. 1 ▸ and 2 ▸).

Finally, the insulated seats/heaters are mounted on the cell body. A specially designed rocking hemisphere enables adjustment of the tilt of the seat on the piston side of the cell. On the cylinder side, a three-screw system allows the translational alignment of the seats.

For the high *P*–*T* synchrotron XRD measurements, a water-cooled system was built to prevent damage to the *xyz* motors, power and thermocouple cables and to enable fast cooling (and hence sample change) at the end of an experiment. The external water-cooling system combined with the thermal insulation of the seats/heaters allows temperatures of 1000 K to be achieved on the sample while the cell body remains at 360 K. High-precision temperature control is achieved remotely from the control room using a dual (2 × 30 V, 2 × 5 A) power supply (TTi EX354RT Triple Power Supply, 300 W). As the number of Mo windings around the WC seats are the same for both heaters, the power consumption and the heat output are initially similar for each heater. About 72 W is necessary to reach a temperature of 1100 K. Throughout the experiments, the cell is continuously flushed with 2 bar s^−1^ of Ar–H_2_ to protect the WC seats and diamond anvils from oxidation. Under such conditions, the heating elements usually last for a minimum of three to four runs at 1000–1100 K, and more for lower temperatures. K-type thermocouples glued onto the diamond anvils, as close as possible to the culet regions, provide real-time monitoring of the temperature with a precision of ±2 K. The differential between the temperature recorded by the thermocouples and the actual sample temperature can be calibrated prior to experiments from the known melting temperatures of native sulfur (388.6 K), NaNO_3_ (581.2 K) and NaCl (1073.7 K) at ambient *P*. Overall, in our tests the difference never exceeded 30 K at the highest *T* conditions. Pressure on the sample can be determined either from the thermal equation of state of standard materials or from the Raman signal of quartz (or zircon), loaded together with the sample, in the high-*P* chamber of the cell (Schmidt & Ziemann, 2000 ▸; Schmidt *et al.*, 2013 ▸). Here, we used NaCl or Au chips loaded together with the sample in the sample chamber (Dorogokupets & Dewaele, 2007 ▸).

### 2.2. *In situ* XRD experiments   

**Figure 2:**
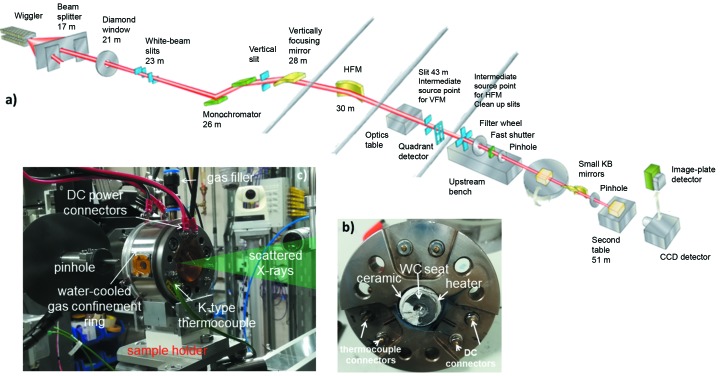
(*a*) Schematic of the I15 beamline at DLS. Photographs of the HXD95 DAC showing (*b*) the hand-made heaters and connections for the power supplies and thermocouples and (*c*) the final setup in position on the sample stage of the XRD station at beamline I15 at DLS.

*In situ* angle-dispersive XRD measurements were made for liquid Ga and PbSiO_3_ glass and melt at up to 3 GPa and 1300 K in the HXD95 DAC at beamline I15 at Diamond Light Source (DLS), UK (Fig. 2 ▸). The focused incident X-ray beam of wavelength λ = 0.2214 Å (56 keV) was collimated using a 70 µm-diameter W-pinhole (20 µm for liquid Ga). Two-dimensional diffraction patterns were collected using a MAR345 CCD detector with acquisition times between 240 s and 600 s.

The HXD95 DAC was equipped with two Boehler-type 500 µm culet diamond anvils. The downstream anvil was partially perforated down to 150 µm in order to minimize background elastic and Compton scattering from the diamond anvils. The powdered starting materials were loaded compactly into a 250 µm pressure chamber drilled into an Re gasket by electrical spark erosion. The cell was continuously flushed with 2 bar s^−1^ of Ar–H_2_ throughout the experiments to protect the cell from oxidation.

XRD patterns were collected under room conditions and high-*P*, and then in 200 K steps up to ∼900 K. From 900 K, XRD patterns were acquired every 5 K to 20 K to ensure precise capture of the melting points. The diffraction patterns were used to monitor both crystallization and melting, with the observation of diffuse rings and corresponding disappearance of crystalline diffraction peaks marking the melting temperature.

The measured diffraction patterns were integrated to one-dimensional profiles using the *DAWN* data analysis suite (Filik *et al.*, 2017 ▸), where the sample-to-detector distance was calibrated using diffraction patterns measured for the LaB_6_ standard.

As a consequence of the applied *P* and *T*, the sample chamber geometry changes during the experiment, as does the environmental contribution to the total scattering; this differs from a PDF experiment performed in a capillary. Therefore, in order to characterize the background scattering arising from the sample environment, additional measurements were collected for the empty cell, before sample loading and after the experimental run, using the gasket recovered from the experiments. The liquid and glass total structure factors *S*(*Q*) were obtained by normalizing the measured diffraction intensities to the *Q*-dependent self-scattering and Compton-scattering components after correcting for background scattering (predominantly Compton scattering from the diamond anvils). The total PDFs *G*(*r*) were obtained from the *S*(*Q*) functions by Fourier transformation. A more detailed description of this structure factor normalization procedure is provided elsewhere (Drewitt *et al.*, 2011 ▸, 2015 ▸).

## 3. Results   

### 3.1. Liquid Ga   

**Figure 3:**
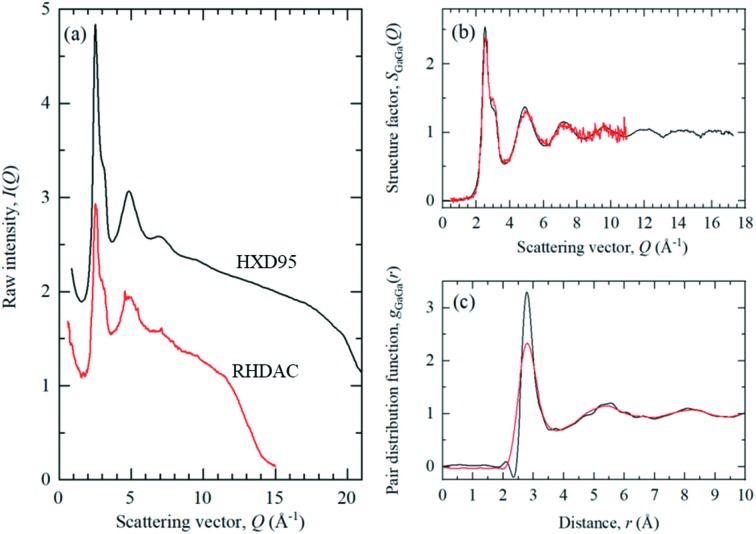
Comparison between the (*a*) measured XRD intensities, (*b*) corresponding partial structure factor *S*_GaGa_(Q) and (*c*) partial PDF *g*_GaGa_(*r*) for liquid Ga at ∼0.1 GPa in the new HXD95 device (black curve) and the externally heated cell (RH-DAC) available on I15 (Drewitt *et al.*, 2018 ▸) (red curve). For clarity, the HXD95 measurement has been displaced vertically.

Gallium is an ideal test case due to its low melting point and negative melting curve, where the crystalline solid melts at ambient *T* under compression (Bosio, 1978 ▸). The measured XRD pattern for liquid Ga in the HXD95 DAC is compared with a previous measurement (Drewitt *et al.*, 2018 ▸) made in an externally heated DAC (Stinton *et al.*, 2014 ▸; Cazorla *et al.*, 2016 ▸; Anzellini *et al.*, 2018 ▸ *a*, 2019 ▸ *b*) at beamline I15 with identical incident beam energy (56 keV), pinhole size (20 µm) and exposure time (240 s) [Fig. 3 ▸(*a*)]. The HXD95 measurement exhibits an improved signal-to-noise ratio, predominantly due to the large sample volume in this cell optimized for measurements at *P* < 5 GPa, compared with the externally heated cell which is optimized for *P*–*T* conditions up to 80 GPa and 900 K. In this monoatomic system, the partial structure factor *S*_GaGa_(*Q*) is measured directly and, aside from the already mentioned improvement in signal-to-noise ratio, the *S*_GaGa_(*Q*) functions measured in the HXD95 and externally heated cells are in general good agreement [Fig. 3 ▸(*b*)]. However, the wider opening of the HXD95, and ability to position the cell close to the detector, also provides a higher maximum accessible scattering vector *Q*_max_ of 17.5 Å^−1^ compared with *Q*_max_ = 11 Å^−1^ for the externally heated cell, resulting in improved resolution in the real-space partial PDF *g*_GaGa_(*r*) [Fig. 3 ▸(*c*)].

### 3.2. Melting point and structure of molten PbSiO_3_   

Pb-silicate glasses have unique optical properties that make them suitable for high-end ‘crystal glass’ tableware, as well as for industrial applications in radiation shielding or in opto­electronic devices (Kohara *et al.*, 2010 ▸). A complete structural description of the PbO–SiO_2_ system by Raman and X-ray absorption spectroscopies can be found in the work by Ben Kacem *et al.* (2017 ▸). These authors showed that the addition of only 5 mol% PbO resulted in a significant decrease of viscosity and that glass transition temperature decreased continuously with increasing PbO contents, concluding that Pb had considerable network-modifying potential even at low concentrations.

**Figure 4:**
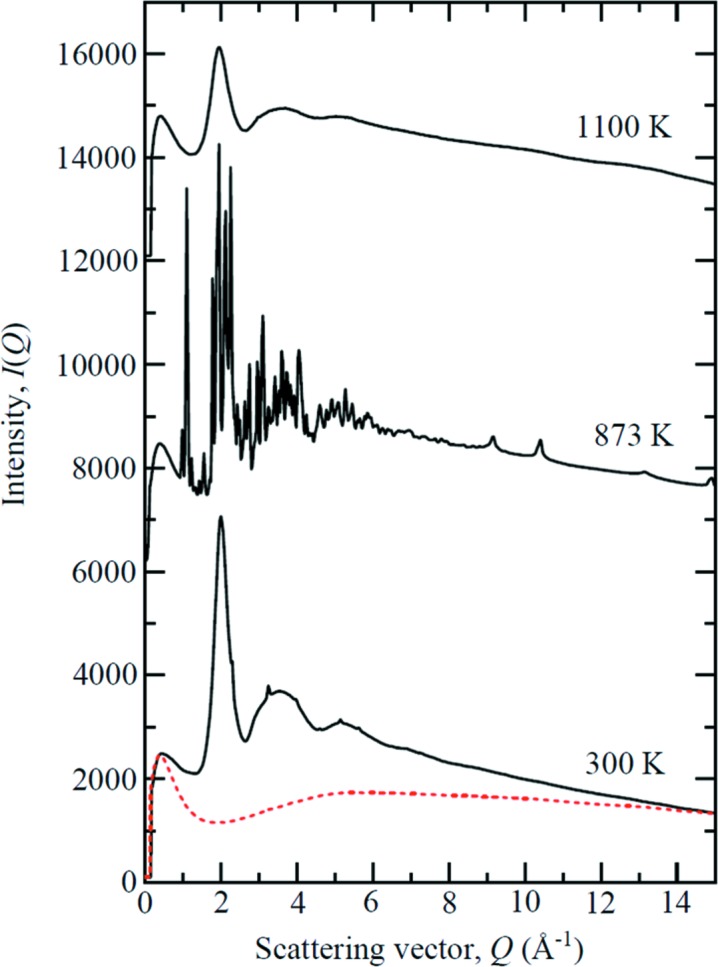
The diffraction patterns for PbSiO_3_ glass measured in the HXD95 showing the glass compressed to 2.5 GPa at ambient *T*, crystallization at 873 K, and the fully molten liquid at 1100 K (solid black curves). The broad peaks in the raw pattern at 873 K for *Q* > 8 Å arise from the diamond anvils. The red dashed curve is the empty cell background measurement. For clarity, the results are displaced vertically.

PbSiO_3_ glass was used to test the capability of the HXD95 DAC at temperatures above 1000 K. The powdered glass was first compressed to 2.5 GPa at room temperature. Under these conditions, the sample exhibits a diffuse diffraction pattern characteristic of amorphous materials (Fig. 4 ▸). At 873 K, the sample is re-crystallized. We have constrained the liquidus of PbSiO_3_ to 1053–1058 K at 2.3 GPa, as determined from the total absence of sharp Bragg reflections. By comparison, melting at room pressure is expected around 973 K (Kaur *et al.*, 2013 ▸).

**Figure 5:**
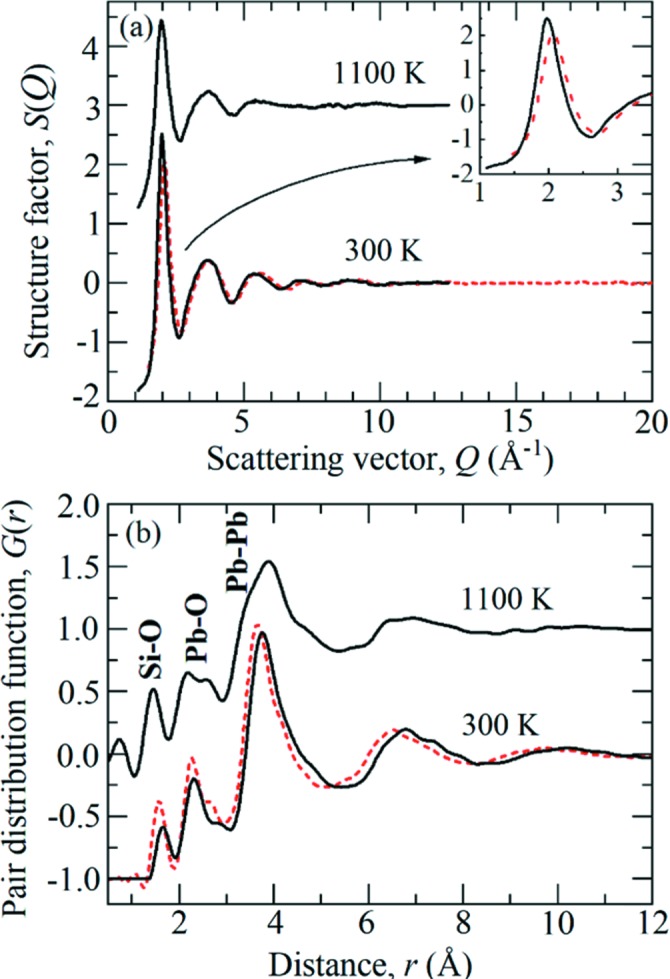
(*a*) The total structure factors *S*(*Q*) for PbSiO_3_ glass and liquid at 2.5 GPa and 300 to 1100 K (solid black curves). The dashed red curves are ambient-*P* measurements of PbSiO_3_ glass made at beamline ID11 at the ESRF. The inset shows comparison between the 300 K measurement at 2.5 GPa and the ambient-*P* glass in the low-*Q* region. (*b*) The total PDFs *G*(*r*) obtained from the corresponding *S*(*Q*) functions in (*a*) by Fourier transformation. For clarity, the results are displaced vertically.

The measured *S*(*Q*) and *G*(*r*) functions for compressed PbSiO_3_ glass and liquid (initial *P* = 2.5 GPa) are shown in Fig. 5 ▸. Subtle changes, including a small shift in the first reciprocal-space peak to lower *Q*, are observed between the *S*(*Q*) measured at 2.5 GPa and the ambient-*P* glass, as measured at beamline ID11 at the European Synchrotron Radiation Facility (ESRF), France. In real-space, the first peak at 1.59 Å corresponds to the nearest-neighbour Si—O bond length. In the ambient-*P* glass, the second peak is split with two nearest-neighbour Pb—O distances at 2.28 and 2.67 Å, consistent with a mixture of fourfold- and sixfold-coordinated Pb—O polyhedra. The high atomic number of Pb means the Pb–Pb correlations receive a substantial weighting, giving rise to a large third peak at 3.66 Å. At 2.5 GPa the Pb—Pb distance increases to 3.76 Å. The high-*P* Si—O and Pb—O peaks experience a degree of broadening and reduction in height; however, this is attributable to a loss in real-space resolution due to the more limited usable *Q*_max_ = 12 Å^−1^ compared with 20 Å^−1^ for the ambient-*P* glass. The high-*P*–*T* liquid *S*(*Q*) is more diffuse than the glass measurement due to the greater degree of structural disorder and liquid dynamics, leading to increased broadening in the Pb—O and Pb—Pb peaks in *G*(*r*). The Pb—O at 2.67 Å becomes more developed at high *T*, indicating an increased fraction of sixfold coordinated Pb—O polyhedra.

## 4. Conclusions and future prospects   

Understanding the atomic-scale structure of molten metals and glasses is a prerequisite for predicting their physico-chemical properties (*e.g.* viscosity, thermal conductivity and diffusivity) and response to different processes. In this paper, we detail the construction of a new modified Bassett-type hydro­thermal diamond-anvil cell, the HXD95, and report example measurements to demonstrate the capability of the device for *in situ* synchrotron XRD measurements of liquids and glasses to 1285 K and 3 GPa.

The modifications applied to this cell allow *in situ* synchrotron XRD experiments to be performed on amorphous materials under extreme conditions of *P* and *T* with an improved signal-to-noise ratio, a maximized *q*-range accessibility and a consequently improved resolution in *r*-space.

This new device has promising applications for studying the crystallization of natural magmas and its effect on melt properties, ultimately affecting eruptive behaviour. To that end, additional developments are still needed to improve the precision of pressure determination to upper crustal and volcanic conditions (*P* < 0.5 GPa). The addition of a Raman spectrometer to the current setup could enable us to use minerals with limited solubility in silicate melts as a more sensitive pressure sensor below 5 kbar [*e.g.* zircon; Schmidt *et al.* (2013 ▸)]. Controlled water fractions, which are necessary to study natural magmas that contain 1–7 wt% depending on composition and *P* and *T* conditions, should be easily achieved, loading starting powders with known OH concentrations [*e.g.* Al(OH)_3_]. The versatility of the HXD95, which also enables the study of aqueous and solute-rich fluids (*e.g.* Louvel *et al.*, 2013 ▸) and is easily amenable to different X-ray techniques that require flexible collection geometries (*e.g. in situ* XAS), opens further opportunities in the fields of materials, environmental or earth sciences. Amongst those, extension to higher *P*–*T* conditions of studies on metal–organic frameworks (Widmer *et al.*, 2019 ▸) could be a promising development.
